# Microarray data and gene expression statistics for *Saccharomyces cerevisiae* exposed to simulated asbestos mine drainage

**DOI:** 10.1016/j.dib.2017.05.021

**Published:** 2017-05-12

**Authors:** Heather E. Driscoll, Janet M. Murray, Erika L. English, Timothy C. Hunter, Kara Pivarski, Elizabeth D. Dolci

**Affiliations:** a Vermont Genetics Network, Department of Biology, Norwich University, 158 Harmon Drive, Northfield, VT 05663, USA; b Vermont Genetics Network, Department of Biology, University of Vermont, 120A Marsh Life Science, Burlington, VT 05405, USA; c Department of Environmental and Health Sciences, Johnson State College, 337 College Road, Johnson, VT 05656, USA; d Advanced Genome Technologies Core, University of Vermont, 149 Beaumont Avenue, Burlington, VT 05405, USA

**Keywords:** VAG, Vermont Asbestos Group, others throughout this manuscript include BAP, biotinylated anti-phycoerytherin antibody, DE, differentially expressed, FDR, false discovery rate, NCBI GEO, National Center for Biotechnology Information Gene Expression Omnibus, NIGMS, National Institute of General Medical Sciences, NIH, National Institutes of Health, PC1, first principal component, PCA, principal components analysis, PGS, Partek® Genomics Suite, RMA, Robust Multichip Average, SAPE, streptavidin-phycoerytherin dye, TKN, Total Kjeldahl Nitrogen, VGN, Vermont Genetics Network, VT-ANR, Vermont Agency of Natural Resource, YPD, yeast extract peptone dextrose, Ultramafic, Chrysotile asbestos, Mine drainage, Gene expression, Microarray, *Saccharomyces cerevisiae*, Environmental toxicology

## Abstract

Here we describe microarray expression data (raw and normalized), experimental metadata, and gene-level data with expression statistics from *Saccharomyces cerevisiae* exposed to simulated asbestos mine drainage from the Vermont Asbestos Group (VAG) Mine on Belvidere Mountain in northern Vermont, USA. For nearly 100 years (between the late 1890s and 1993), chrysotile asbestos fibers were extracted from serpentinized ultramafic rock at the VAG Mine for use in construction and manufacturing industries. Studies have shown that water courses and streambeds nearby have become contaminated with asbestos mine tailings runoff, including elevated levels of magnesium, nickel, chromium, and arsenic, elevated pH, and chrysotile asbestos-laden mine tailings, due to leaching and gradual erosion of massive piles of mine waste covering approximately 9 km^2^. We exposed yeast to simulated VAG Mine tailings leachate to help gain insight on how eukaryotic cells exposed to VAG Mine drainage may respond in the mine environment. Affymetrix GeneChip® Yeast Genome 2.0 Arrays were utilized to assess gene expression after 24-h exposure to simulated VAG Mine tailings runoff. The chemistry of mine-tailings leachate, mine-tailings leachate plus yeast extract peptone dextrose media, and control yeast extract peptone dextrose media is also reported. To our knowledge this is the first dataset to assess global gene expression patterns in a eukaryotic model system simulating asbestos mine tailings runoff exposure. Raw and normalized gene expression data are accessible through the National Center for Biotechnology Information Gene Expression Omnibus (NCBI GEO) Database Series GSE89875 (https://www.ncbi.nlm.nih.gov/geo/query/acc.cgi?acc=GSE89875).

**Specifications table**

**Table:** 

Subject area	*Biology*
More specific subject area	*Transcriptomics, environmental toxicology*
Type of data	*Data tables and corresponding visualizations*
How data was acquired	*Affymetrix GeneChip® Yeast Genome 2.0 Array, Robust Multichip Average normalization, statistical analysis*
Data formats	*Raw and normalized microarray data table* *Water and media chemistry metadata table* *One-way analysis of variance results gene table* *Differentially expressed gene table*
Experimental factors	*Treatment (asbestos mine tailings-exposed) vs. control*
Experimental features	*Total RNA was recovered from four Saccharomyces cerevisiae strain NRRLY-12632 (ATCC® 18824) replicates exposed to simulated asbestos mine tailings runoff for a 24-h period and three control replicates.*
Data source location	*Lowell tailings pile, Vermont Asbestos Group Mine, Lowell, Vermont, USA (near 44°45׳51.8"N 72°31׳22.2"W)*
Data accessibility	*Microarray data and some sample metadata are available at NCBI GEO Series*GSE89875*: r*https://www.ncbi.nlm.nih.gov/geo/query/acc.cgi?acc=GSE89875. *Water and media chemistry data tables as well as gene statistical data tables are available in this article.*

**Value of the data**

• This is the first global gene expression dataset from a eukaryotic model system exposed to asbestos mine tailings runoff.
• Water chemistry of the simulated asbestos mine-tailings leachate used in this experiment is geochemically similar to runoff from the Vermont Asbestos Group mine and may well represent physio-chemical properties of other alkaline mine drainage systems.
• Our thorough metadata descriptions, including water and growth media chemistry, maximize the utility of these microarray data to others studying the biological effects of asbestos mine drainage.
• Both the normalized intensity data and gene expression profile data can be used in functional analysis and systems biology approaches to understanding complex biological processes affected by asbestos mine runoff.

## 1 Data

### 1.1 Water and media chemistry data

Simulated asbestos mine drainage water chemistry shows similarities to the geochemistry of surface water samples collected previously from the VAG Mine by the Vermont Agency of Natural Resources (VT-ANR), particularly pH and conductivity for simulated asbestos mine drainage and impaired site Burgess Branch 5.0 [1]. Table 1 includes select chemical properties from simulated asbestos mine drainage from this research as well as four localities from the VT-ANR report [1], including Hutchins Brook 3.1 and Burgess Branch Tributary 8, two control sites that do not receive asbestos mine drainage runoff and two asbestos mine drainage-impaired sites downstream of the mine, Hutchins Brook 2.1 and Burgess Branch 5.0. These results establish the mine-tailings leachate prepared in the lab for this study as a reasonable substitute for VAG Mine drainage. Complete water chemistry for this study are available in Supplementary Table 1.

**Table 1:** Select parameters from simulated asbestos mine drainage water chemistry from this study compared to previously analyzed environmental samples from VT-ANR water quality reports/surface water analyses [1].

**Parameter**	**Units**	**Simulated asbestos mine drainage prepared on two dates in 2014 (Spring/Summer)**	**Burgess Br. Trib. 8 (control)** **2007**	**Burgess Br. 5.0** **(impaired)** **2007 2009**		**Hutchins Br. 3.1** **(control)** **2007**	**Hutchins Br. 2.1** **(impaired)** **2007 2009**	
pH		9.2/-	7.8	**8.53**	8.39	7.65	8.44	8.04
Alkalinity	mg/L	-/-	43.5	**246**	**276**	48.8	**134**	**95.3**
Conductivity	umho	-/541	98.3	**495**	**534**	103	**278**	186
Mg^2+^	mg/L	120/120	6.61	**59.5**	**67.8**	7.78	**32.7**	**20.9**
Fe^2+/3+^	μg/L	<20/<20	129	<50	103	<50	<50	54.3
Ca^2+^	mg/L	1.3/1.4	7.27	13.8	15.6	6.76	8.43	7.64
SO_4_^2−^	mg/L	-/2.7	5.08	**22.8**	**24.6**	3.97	10.7	6.47
Cl^−^	mg/L	-/<2.5	<2	3.31	3.5	<2	<2	<2
Ni^2+^	μg/L	<5/<5	<5	**16.6**	**15.4**	<5	**73.9**	**38.7**
Mn^2+^	μg/L	<20/<20	21.2	24.4	**76.6**	<5	<5	5.05
K^+^	mg/L	<0.5/<0.5	0.35	1.2	1.36	0.14	0.34	0.25
Na^+^	mg/L	<0.5/<0.5	1	2.47	2.89	0.43	0.73	0.53
As	μg/L	<1/<1	1	2.7	3.1	0.86	**12.2**	3.13

Values in bold indicate large differences between impaired and control sites in the VT-ANR report.

Control group media chemistry is notably different than simulated asbestos mine drainage media (Table 2). Total magnesium concentration is considerably higher in simulated asbestos mine drainage than control media (100 mg/L and 2.6 mg/L, respectively). Additionally, the pH of the experimental group media suggests that simulated asbestos mine drainage water (pH 9.2) was buffered by the yeast extract peptone dextrose (YPD) media (pH 7.23).

**Table 2:** Complete chemistry from simulated asbestos mine drainage media and control media.

**Parameter**	**Simulated asbestos mine drainage media**	**Control media**	**Units**	**Method**
pH	7.23	6.38		
Conductivity	2780	2490	umhos/cm	EPA 120.1
Aluminum, total	<0.040	<0.040	mg/L	EPA 200.7
Antimony, total	<0.002	<0.002	mg/L	SM20 3113B
Arsenic, total	0.001	<0.001	mg/L	SM20 3113B
Beryllium, total	<0.002	<0.002	mg/L	EPA 200.7
Bromide	<25	<25	mg/L	EPA 9056A
Cadmium, total	<0.004	<0.004	mg/L	EPA 200.7
Calcium, total	7.1	5.7	mg/L	EPA 200.7
Chloride	330	210	mg/L	EPA 300.0
Chromium, total	<0.010	<0.010	mg/L	EPA 200.7
Cobalt, total	<0.040	<0.040	mg/L	EPA 200.7
Copper, total	<0.040	<0.040	mg/L	EPA 200.7
Iron, total	0.47	0.3	mg/L	EPA 200.7
Lead, total	<0.001	<0.001	mg/L	SM20 3113B
Magnesium, total	100	2.6	mg/L	EPA 200.7
Manganese, total	<0.040	<0.040	mg/L	EPA 200.7
Mercury, total	<0.0002	<0.0002	mg/L	EPA 245.1
Molybdenum, total	<0.040	<0.040	mg/L	EPA 200.7
Nickel, total	<0.010	<0.010	mg/L	EPA 200.7
Nitrate as N	2.9	<2.0	mg/L	EPA 300.0
Nitrite as N	<2.0	<2.0	mg/L	EPA 300.0
Phosphorus, total	190	180	mg/L	EPA 6010C
Potassium, total	510	490	mg/L	EPA 200.7
Selenium, total	<0.002	<0.002	mg/L	SM20 3113B
Silver, total	<0.040	<0.040	mg/L	EPA 200.7
Sodium, total	300	290	mg/L	EPA 200.7
Sulfate	160	100	mg/L	EPA 300.0
Thallium, total	<0.001	<0.001	mg/L	SM20 3113B-04
Tin, total	<0.040	<0.040	mg/L	EPA 200.7
Total Kjeldahl nitrogen (TKN)	840	890	mg/L	EPA 351.2, R.2
Zinc, total	0.96	0.93	mg/L	EPA 200.7

### 1.2 Microarray data

**Fig. 1:**
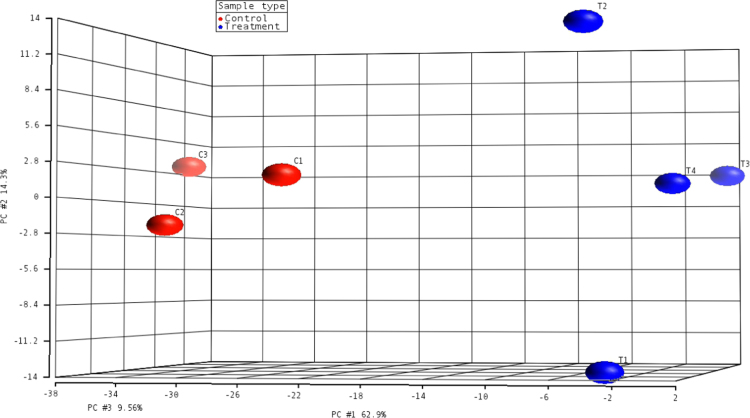
Principal components analysis plot based on RMA-normalized *S. cerevisiae*-only intensity data from GeneChip® Yeast Genome 2.0 Arrays.

**Fig. 2:**
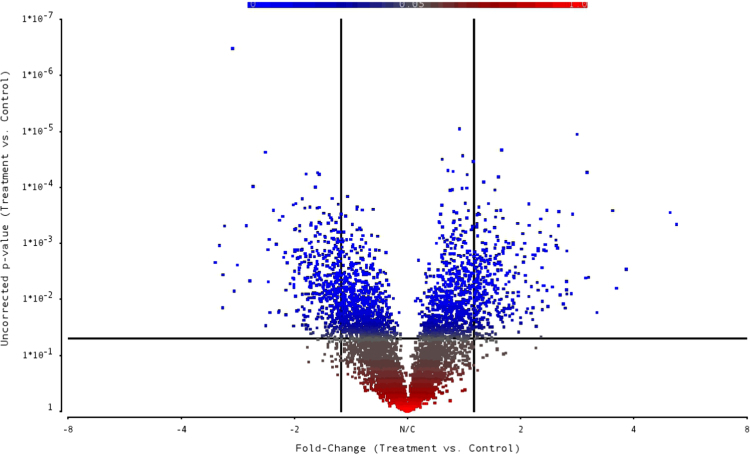
Volcano plot containing all genes from the one-way ANOVA based on *S. cerevisiae*-only probeset IDs (5716). Cutoff lines set to a fold change >|1.5| (*x*-axis) and uncorrected *p*-value <0.05 (*y*-axis).

**Fig. 3:**
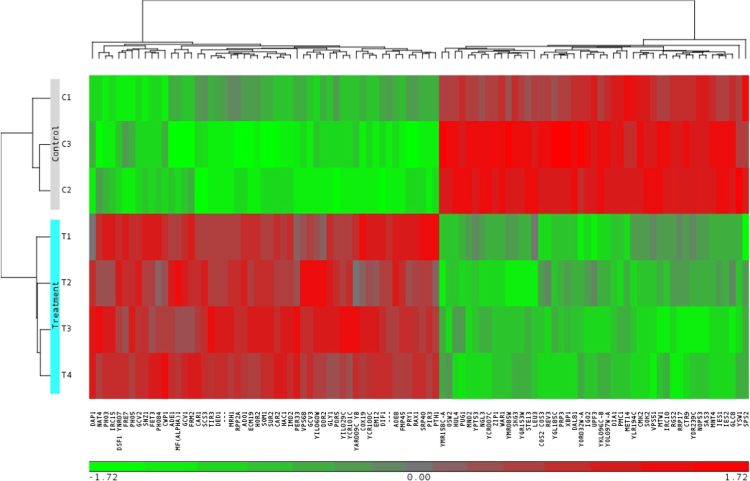
Heatmap of the genes that passed a conservative filtering threshold of FDR 0.05 and a fold change >|1.9| (100 genes). The RMA-normalized expression data used as input for this analysis were standardized with a mean of zero and a standard deviation of one.

Raw and normalized microarray data for all Affymetrix GeneChip® Yeast Genome 2.0 Array probesets are available through NCBI GEO Series GSE89875: https://www.ncbi.nlm.nih.gov/geo/query/acc.cgi?acc=GSE89875. Microarray data exploration at the sample-level using principal components analysis (PCA) identifies distinct molecular phenotypes in simulated asbestos mine drainage versus control samples and shows no significant outliers in the study set (Fig. 1). The first principal component (PC1) correlates with our single biological factor, simulated asbestos mine drainage exposure. Gene-level ANOVA with *S. cerevisiae*-only probesets found 735 genes that are differentially expressed between treatment and control groups (unadjusted *p*-value <0.05; fold change >|1.5|) (Supplementary Tables 2–3). Of these 735 genes, 404 genes are down-regulated and 331 genes are up-regulated (Fig. 2). Of the 761 genes that passed a more conservative false discovery rate (FDR) filtering threshold of 0.05, 405 exhibited a fold change >|1.5| (“step-up” *p*-value, Supplementary Tables 2–3, column K). A hierarchical clustering map includes the highest and lowest expressers (FDR <0.05; fold change >|1.9|) for treatment and control groups (Fig. 3).

## 2 Experimental design, materials and methods

### 2.1 Mine tailings collection and processing

**Fig. 4:**
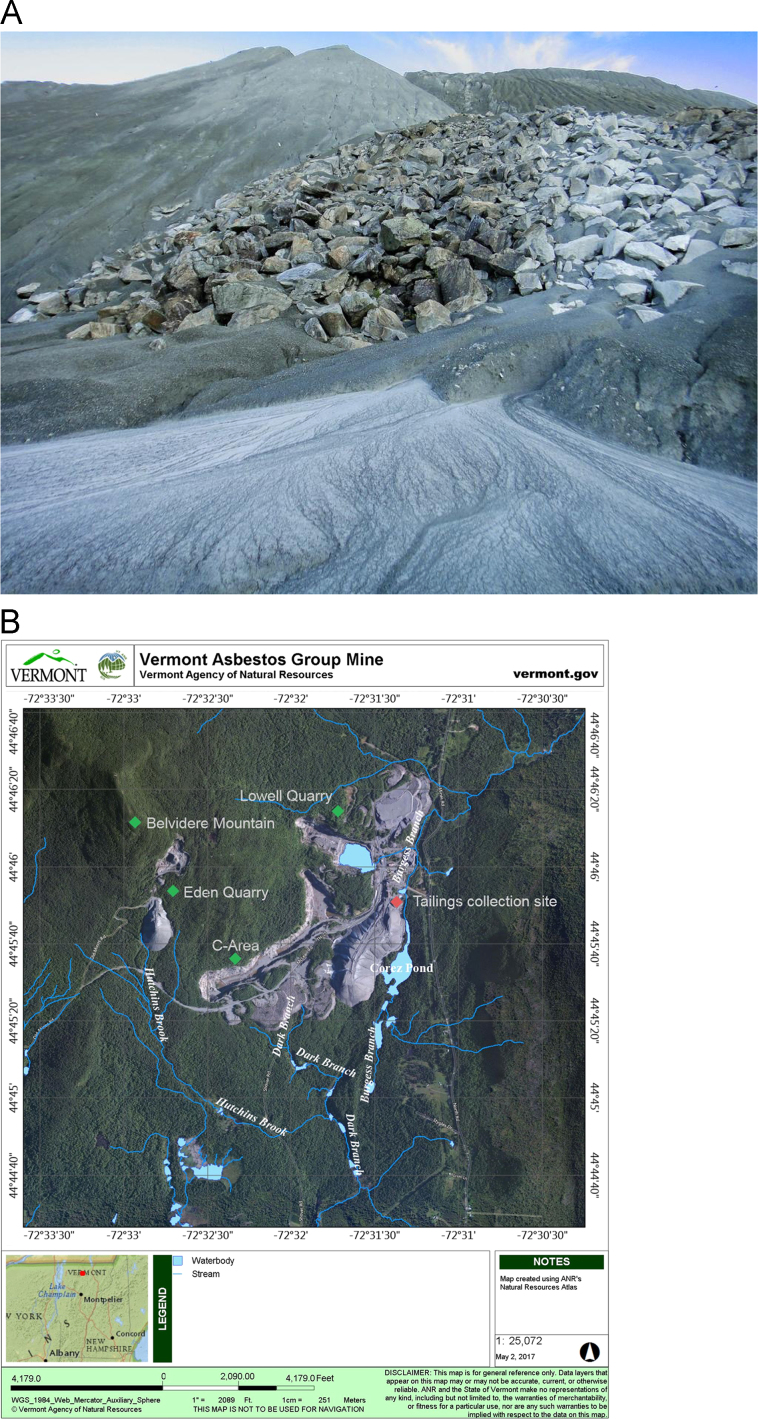
A. Vermont Asbestos Group Mine waste and tailings piles with runoff (photo source: Tony Rich @Asbestorama on Flickr, www.flickr.com/photos/asbestos_pix/). B**.** Aerial map marked with VAG Mine quarries and tailings collection site. Drainage from the VAG Mine enters two Lake Champlain watersheds, Hutchins Brook, which runs into the Lamoille River watershed to the South, and Burgess Branch, which runs into the Missisquoi River watershed to the North. Map source is http://anrmaps.vermont.gov/websites/anra/.

A composite sample of the mine tailings surface was collected on June 19, 2013 from the Lowell Quarry at the VAG mine, Lowell, Vermont, USA (near 44°45′51.8″N 72°31′22.2″W) shown in Fig. 4. The tailings were collected in sterile Whirl-pak® bags (Nasco, Fort Atkinson, Wisconsin, USA) and stored at 4 °C until use. Tailings were processed and the leachate was prepared in the Dolci Laboratory at Johnson State College (Johnson, Vermont, USA) in February 2014. A 250 g sample of tailings was mixed with an equal volume (250 ml) of deionized water followed by exposure to three freeze–thaw cycles (−80 °C) over ten days. The thaw cycle was performed at room temperature with gentle rocking. Water was retrieved from settled tailings followed by filtration through a 0.22 µm polycarbonate filter. Modified YPD growth media was prepared with either simulated asbestos mine drainage or deionized water as follows: 12.5 g YPD powder (Fisher Scientific catalog #BP2469), 10 g glucose, 500 ml water and autoclaved [2]. Note, mineralogical characterization of VAG mine tailings was not performed for this study, however, it has been shown previously that magnesium, silicon, iron, and aluminum are major constituents and nickel and chromium are minor constituents of the tailings [3], [4].

### 2.2 Physio-chemistry methods for water and media

Samples of the simulated asbestos mine drainage water (150 ml) as well as prepared simulated asbestos mine drainage media and control media (500 ml each) were sent to Endyne Inc. (Williston, Vermont, USA) for analysis. The methodology for the water chemical analyses reported in Table 1 and the complete water chemistry analyses can be found in Supplementary Table 1. Complete media chemistry methods are reported in Table 2.

### 2.3 Strain and growth conditions

A 48-h culture of *S. cerevisiae* strain NRRL Y-12632 (ATCC® 18824) grown in the control YDP media (described above) was used as inoculum for seven independent sample cultures, four experimental and three control. Growth was monitored by measuring the absorbance, A, at 600 nm (A_600_) and samples were inoculated into either fresh YPD control media or YPD simulated asbestos mine drainage media (A_600_ 0.05). The cultures were incubated aerobically (225 rpm) at 30 °C for 24 h, at which point cells from each sample were harvested (1.5 ml of culture media) and pelleted by centrifugation at 4000×*g* (6800 rpm) for RNA isolation.

### 2.4 RNA isolation and target preparation

Total RNA isolation for all samples was performed as described in [2]. Briefly, to form spheroplasts, all samples were treated with lyticase (10U/μl). Guanidinium isothiocyanate contained in the RLT buffer from the RNeasy® Plus Mini kit (Qiagen, Valencia, California, USA) was used to rupture cell membranes. Total RNA was collected using silica gel spin columns and DNase I was added to degrade genomic DNA. A Nanodrop 1000 Spectrophotometer (Thermo Scientific, Madison, Wisconsin, USA) was used to measure the quantity and quality of the resulting total RNA. Additionally, RNA quality was assayed using the Agilent BioAnalyzer (Agilent Technologies, Santa Clara, California, USA). No samples were excluded due to low RNA quality.

Total RNA input of 50 ng was used to generate cDNA with the Ovation® Pico WTA System V2 kit (NuGEN Technologies Inc., San Carlos, California, USA). cDNA samples were then purified using an Agencourt® RNAClean XP magnetic bead protocol (Beckman Coulter Inc., Brea, California, USA). Following purification, samples were amplified using SPIA reagents from the Ovation® Pico kit. A final cDNA purification was performed using an Agencourt® RNAClean XP magnetic bead protocol. Sample concentrations were determined using a 33 μg/ml/A260 constant on a Nanodrop 1000 Spectrophotometer. Approximately 4 μg of cDNA was fragmented and labeled with the NuGEN Encore® Biotin Module. Efficiency of the biotin labeling reaction was verified using NeutrAvidin (10 mg/ml) with a gel-shift assay.

A hybridization mixture with controls, including control B2 oligo, 20× eukaryotic control mix, herring sperm, acetylated BSA, 2× hybridization buffer and water, was added to all seven samples. 90 μl of this mixture was injected into a GeneChip® Yeast Genome 2.0 Array (Affymetrix, Santa Clara, California, USA) and placed in the Affymetrix GeneChip® Hybridization Oven 640 at 45 °C and 60 rpm for 18 h overnight. A staining mixture containing streptavidin-phycoerytherin dye (SAPE) and biotinylated anti-phycoerytherin antibody (BAP) was added to amplify signal intensities. Arrays were stained and washed in the Affymetrix GeneChip® Fluidics Station 450 using the Mini_euk2V3_450 fluidics script. All arrays were scanned with the Affymetrix GeneChip® Scanner 3000 and raw analysis performed with Affymetrix Expression Console^TM^ software. The raw data images produced from the scanner were processed into CEL files, which contained measure intensities for each probe on the array.

### 2.5 Gene expression statistical methods

Seven CEL files (four experimental and three control) were imported into the Gene Expression Workflow in Partek® Genomics Suite (PGS) version 6.6 (Partek Inc., St. Louis, MO, USA, www.partek.com). Background correction, quantile normalization, log2 transformation, and probeset summarization were performed using default settings for the Robust Multichip Average (RMA) procedure [5], [6], [7]. After *Schizosaccharomyces pombe* probesets, also present on the GeneChip® Yeast Genome 2.0 Array, were removed, a PCA was performed using a covariance dispersion matrix as part of the quality control of the data.

Differential expression (DE) between yeast exposed to simulated asbestos mine drainage (experimental) and unexposed yeast (control) was predicted at the gene-level (probesets summarized into transcript clusters/genes). One-way analysis of variance (ANOVA) was used to compare the individual gene expression data with respect to mine-tailings exposure, i.e. simulated asbestos mine drainage versus control. DE genes were defined based on an absolute fold change equal to or greater than 1.5 and an unadjusted *p*-value <0.05. Multiple comparison correction was performed using a FDR of 0.05 [8]. Input for the heatmap was the RMA-normalized expression data standardized to a mean of zero and a standard deviation of one for the subset of DE genes with an absolute fold change equal to or greater than 1.9 and a FDR <0.05.

## Appendix A Supplementary material

Supplementary Tables 1–3 file. An Excel file containing water chemistry methods and results for simulated asbestos mine drainage water, complete gene-level ANOVA results for S. cerevisiae-only probesets, and a subset of genes from the ANOVA results, which have an unadjusted *p*-value <0.05 and a fold change >|1.5|. The latter are considered differentially expressed (DE) between experimental and control groups in this study. Abbreviations: Probeset ID, Affymetrix GeneChip® Yeast Genome 2.0 Array probeset identifier; SGD, Saccharomyces Genome Database; stepup *p*-value, Benjamini & Hochberg׳s false discovery rate. Supplementary material
